# Effects of Expiratory Muscle Strength Training on Swallowing in Survivors of Critical Illness: A Protocol for a Systematic Review and Meta‐Analysis

**DOI:** 10.1002/hsr2.70337

**Published:** 2025-02-03

**Authors:** Philip Skurok, Brian W. Johnston, Emma Brown, Caroline Timothy, Christopher Morse, Peter Turton

**Affiliations:** ^1^ Intensive Care Unit Warrington and Halton Hospitals NHS Trust Warrington UK; ^2^ Liverpool Centre for Cardiovascular Sciences University of Liverpool Liverpool UK; ^3^ Department of Cardiovascular and Metabolic Medicine, Institute of Life Course and Medical Sciences University of Liverpool Liverpool UK; ^4^ Department of Sport and Exercise Sciences, Institute of Sport Manchester Metropolitan University Manchester UK

**Keywords:** critical care, dysphagia, expiratory muscle training, extubation, mechanical ventilation, respiratory muscle training

## Abstract

**Background and Aims:**

Post Extubation Dysphagia (PED) is a common consequence of mechanical ventilation. Muscular weakness and atrophy are potential causes. Expiratory Muscle Strength Training (EMST) is a technique whereby a subject exhales against a resistance, strengthening the muscles of expiration. There is evidence that EMST causes activation and hypertrophy of the muscles of swallowing, with clinical evidence that it improves swallowing in certain populations. The aim of this systematic review is to collate the existing literature concerning evaluation of swallowing after extubation, and whether EMST positively affects these measures.

**Methods:**

We will perform a systematic review of the literature by searching electronic databases (Pubmed, Medline, EMBASE, and the Cochrane Library), for articles where EMST has been performed (alone or in conjunction with inspiratory muscle training), in patients who have been liberated from a period of mechanical ventilation. We will identify studies that evaluate swallowing after extubation, listing the methods used to evaluate swallowing and data will be extracted from studies evaluating the impact EMST has on these measures.

**Results:**

We will undertake meta‐analysis if data permits. Risk of bias will be assessed using the Risk of Bias 2 tool or the Newcastle Ottawa Score for randomized and non‐randomized trials. We will use The Grading of Recommendations Assessment, Development and Evaluation (GRADE) approach to assess the quality of evidence.

**Conclusion:**

The results of this systematic review will enable us to assess the current literature on the use of EMST in critical care, and whether the intervention improves swallowing and respiratory outcomes.

**Trial Registration:** *PROSPERO registration*: 42023444479.

## 1 Introduction

Dysphagia is a common consequence of prolonged mechanical ventilation during critical illness [1]. A recent meta‐analysis estimated the incidence of Post‐Extubation Dysphagia (PED) at 41%, and of those with PED, 36% suffer from silent aspiration [2], with older patients at higher risk of delayed resolution of their swallowing impairment after aspiration [3].

PED is defined as “the inability to effectively transfer food from the mouth into the stomach” after a period of mechanical ventilation [4]. A step‐wise approach to it's diagnosis has been suggested in critically ill patients, consisting of initial screening, bedside assessment by a specialist, and confirmatory testing [5]. The Water Swallowing Test (WST) is an example of one such screening test, and is performed by allowing a patient to attempt to swallow water, while observing for evidence of coughing. One observational study demonstrated that 87.5% of patients with a positive WST went on to have dysphagia confirmed by specialists [6]. The diagnosis can be confirmed by either Flexible Endoscopic Evaluation of Swallowing (FEES), or by Videofluroscopic Swallowing Studies (VFSS) [7]. In addition to WST, a number of other noninstrumental tests have been devised [8], but few of these methods have been validated specifically for intensive care patient populations [5]. Although tools have been validated in intensive care patients [9], it is recognized that there are currently no standardized assessment guidelines [10].

The causes of PED are often multifactorial, including direct trauma from the endotracheal tube, loss of oropharyngeal sensation, and impaired neuromuscular function [11]. Muscle atrophy during critical illness is well described, affecting muscles of the limbs [12], trunk [13, 14], and respiratory system [15, 16]. Such atrophy will result in reductions in muscular strength that may impact swallowing and secretion clearance post extubation.

Respiratory muscle training is a technique whereby the respiratory muscles are strengthened via the application of resistance during either inspiration or expiration. In critically ill patients, much work has focussed on Inspiratory Muscle Training (IMT), where the inspiratory muscles (namely, the diaphragm) are trained by applying resistance during inspiration [17]. Such training has been shown to increase inspiratory muscle strength [18], quality of life [19], and shorten weaning times from mechanical ventilation [20].

Expiratory muscle strength training (EMST) applies resistance during expiration. In healthy volunteers, exhaling against increasing resistance leads to activation of the abdominal oblique muscles [21] and *rectus abdominis* [22]. EMST has also been shown to increase the thickness of the abdominal muscles [23]. However, the effects of EMST are not limited to expiratory musculature. Surface electromyography has demonstrated that the muscles of swallowing exhibit electrical activation during EMST [24], and leads to specific hypertrophy of the *geniohyoid* muscle, in addition to increased expiratory muscle strength [25]. It is therefore suggested that the known effects of EMST on the muscles of swallowing could be a potential therapeutic target after extubation.

The functional effects of EMST have been described in patients with neurological and respiratory disorders [26]; for example, EMST has been shown in a randomized control trial to improve dysphagia severity in patients with Parkinson's disease [27], and improved some swallowing outcomes in patients with sub‐acute stroke [28]. However a systematic review of 11 studies found that EMST had variable effects on measures of swallowing, with studies covering a variety of aetiologies and methods to evaluate swallowing [29]. Of the studies listed in the review, none focussed on patients with PED.

We hypothesize that in comparison to other aetiologies, there is much less evidence available on the effects of EMST in survivors of critical illness, and the aim of this systematic review is to establish what is currently known about the use and effects of EMST in these patients.

### 1.1 Objective of the Systematic Review

To conduct a systematic review and, where data allow, a meta‐analysis of the literature to assess the effects of EMST (either alone or in conjunction with IMT) on swallowing, in survivors of critical illness.

## 2 Methods and Analysis

This protocol describes the search strategy, study selection, inclusion criteria, data extraction and analysis for a systematic review of expiratory muscle strength training in survivors of critical illness and is reported in accordance with the Preferred Reporting Items for Systematic Review and Meta‐Analysis Protocols (PRISMA‐P) [30].

Ethical approval is not required for a systematic review protocol, and this protocol is registered with the International Prospective Register of Systematic Reviews (PROSPERO) CRD: 42023444479.

### 2.1 Search Strategy

We will engage the services of specialist healthcare research librarians and conduct a comprehensive systematic search of academic databases including:

1) Medline.

2) PubMed.

3) Embase.

4) Cochrane (Reviews and CENTRAL).

5) CINAHL.

Medical subject headings (MEsH) will be utilized. We will review bibliographies of included studies for any additional results that meet the inclusion criteria. We will search all relevant trial registries and perform searches of any relevant gray literature.

A full description of our search strategy is included in Table 1 but will focus on the population (critically ill patients), participants (patients that have survived a critical care admission and required mechanical ventilation for > 48 h), and the intervention (expiratory muscle strength training). Based on preliminary searches, we anticipate a limited number of eligible papers. Therefore, we would broaden the search terms to include a wider range of papers.

**Table 1:** Search strategy.

Database	Search terms
Medline	(MH “critical care”) OR (MH “intensive care units”) OR (AB “critical care”) OR (AB “intensive care”) OR (AB ICU) OR (AB “mechanical* ventilat*”) AND (AB “muscle strength training”) OR (AB “muscle training”) OR (AB “muscle strength”) OR (AB “respiratory training”) OR (AB “respiratory muscle*”) OR (MH “Respiratory Muscles”) AND (TX expirat*) AND (MH deglutition) OR (MH “deglutition disorders”) OR (AB swallow*) OR (AB dysphagia) OR (AB deglutition)
CINAHL	(MH “critical care”) OR (MH “intensive care units”) OR (AB “critical care”) OR (AB “intensive care”) OR (AB ICU) OR (AB “mechanical* ventilat*”) AND (AB “muscle strength training”) OR (AB “muscle training”) OR (AB “muscle strength”) OR (AB “respiratory training”) OR (AB “respiratory muscle*”) OR (MH “Respiratory Muscles”) AND (TX expirat*) AND (MH deglutition) OR (MH “deglutition disorders”) OR (MH “swallowing therapy”) OR (AB swallow*) OR (AB dysphagia) OR (AB deglutition)
Cochrane Reviews	(MeSH “critical care”) OR (MeSH “intensive care units”) OR (“critical care” ti,ab,kw) OR (“intensive care” ti,ab,kw) OR (ICU ti,ab,kw) OR (mechanical* NEXT ventilat*ti,ab,kw) AND (“muscle strength training” ti,ab,kw) OR (“muscle training” ti,ab,kw) OR (“muscle strength” ti,ab,kw) OR (“respiratory training” ti,ab,kw) OR (respiratory NEXT muscle*ti,ab,kw) OR (MeSH “Respiratory Muscles”) AND (expirat*) AND (MeSH deglutition) OR (MeSH “deglutition disorders”) OR (swallow* ti,ab,kw) OR (dysphagia ti,ab,kw) OR (deglutition ti,ab,kw)
Cochrane CENTRAL	MeSH “critical care”) OR (MeSH “intensive care units”) OR (“critical care” ti,ab,kw) OR (“intensive care” ti,ab,kw) OR (ICU ti,ab,kw) OR (mechanical* NEXT ventilat*ti,ab,kw) AND (“muscle strength training” ti,ab,kw) OR (“muscle training” ti,ab,kw) OR (“muscle strength” ti,ab,kw) OR (“respiratory training” ti,ab,kw) OR (respiratory NEXT muscle*ti,ab,kw) OR (MeSH “Respiratory Muscles”) AND (expirat*) AND (MeSH deglutition) OR (MeSH “deglutition disorders”) OR (swallow* ti,ab,kw) OR (dysphagia ti,ab,kw) OR (deglutition ti,ab,kw)
Embase	(intensive care unit/) OR (“critical care”.ab) OR (“intensive care”.ab) OR (ICU.ab) OR (“mechanical* ventilat*”.ab) AND (“muscle strength training”.ab) OR (“muscle training”.ab) OR (“muscle strength”.ab) OR (“respiratory training”.ab) OR (“respiratory muscle*”.ab) OR (breathing muscle/) AND (expirat*.af) AND (swallowing/) OR (swallowing reflex/) OR (dysphagia/) OR (swallow*.ab) OR (dysphagia.ab) OR (deglutition.ab)
PubMed	(“critical care”[MeSH Terms]) OR (“intensive care” [MeSH Terms]) OR (“critical care”[Title/Abstract]) OR (“intensive care”[Title/Abstract]) OR (ICU[Title/Abstract]) OR (“mechanical* ventilat*”[Title/Abstract]) AND (“muscle strength training”[Title/Abstract]) OR (“muscle training”[Title/Abstract]) OR (“muscle strength”[Title/Abstract]) OR (“respiratory training”[Title/Abstract]) OR (“respiratory muscle*”[Title/Abstract]) OR (“Respiratory Muscles” [MeSH Terms]) AND (expirat*[Text Word]) AND (deglutition[MeSH Terms]) OR (“deglutition disorders”[MeSH Terms) OR (deglutition[Title/Abstract]) OR (swallow*[Title/Abstract) OR (dysphagia[Title/Abstract)

### 2.2 Inclusion and Exclusion Criteria

We will include all quantitative studies published in English since 2000.

#### 2.2.1 Study Design

We anticipate that there will be limited studies therefore we will not limit the included studies by study design. We will include relevant studies that meet our inclusion criteria including case‐series, observational studies, cohort studies, randomized control trials, systematic reviews, and meta‐analyses. We will review the reference lists of all included studies in systematic reviews and meta‐analyses and include additional studies if they meet the inclusion criteria.

#### 2.2.2 Population

Papers that include adult patients (greater than 18 years old) admitted to a critical care setting and underwent mechanical ventilation for greater than 48 h will be included. All patients will have been liberated from mechanical ventilation (i.e. they have no airway devices in situ) and are performing EMST using training devices applied directly to the mouth.

### 2.3 Type of Intervention and Comparators

#### 2.3.1 Intervention

The review will include studies that evaluate EMST alone or in conjunction with IMT in patients who are survivors of critical illness. This includes training using mechanical (e.g., spring loaded) or electronic devices that are applied directly to a patient's mouth via a mouthpiece. We will extract and state the training techniques employed in each study. Training regimes are usually defined by a patient performing a set number of repetitions per day, with the training load set at a specific percentage of the patient's own Maximal Expiratory Pressure (MEP) [29].

#### 2.3.2 Comparators

The review will include studies that employ sham muscle strength training programs as controls, for example where the training load is set to a negligible percentage of the individual patient's own Maximal Expiratory Pressure. We will also include studies where the control groups are randomized to routine or standard care with no sham treatment. It is recognized that the protocol design includes observational studies where there is no comparator group.

#### 2.3.3 Exclusion Criteria

We will exclude studies that only report data in (1) patients that remained intubated for less than 48 h, (2) patients with spinal injuries requiring mechanical ventilation, (3) patients in long‐term ventilatory support centres (including continuous positive pressure ventilation and ventilation via tracheostomy), (4) patients with a diagnosis of acute or chronic neuromuscular disorders, (5) patients that are pregnant, (6) patients with known head and neck cancers.

### 2.4 Outcome Measures

#### 2.4.1 Primary Outcome Measure

Our primary outcome of interest is the measures used to evaluate swallowing function as defined by the authors of included studies.

We anticipate heterogeneity in the evaluation measures used therefore we will extract data on the use of bedside screening tests, clinical evaluations of swallowing, and instrumental methods.

Where instrumental methods are used, we will detail test‐in protocols used in each study.

We will extract data on the effectiveness of each method of swallowing evaluation, we will also extract the measurement tools used. If data allow we will group and combine the quantitative data and if sufficient meta‐analysis on the effectiveness of the measure of swallowing function.

#### 2.4.2 Secondary Outcome Measures

Secondary outcome measures will include:

1) Any measure of cough function, including but not limited to peak cough flow rates (L/min).

2) Changes in Maximal Inspiratory Pressure (MIP) and Maximal Expiratory Pressure (MEP, both in cm H_2_O).

3) Expiratory muscle thickness on ultrasound, specifically external and internal obliques, rectus abdominus and infra/suprahyoid muscles (mm).

4) Incidence of aspiration as identified during evaluation of swallowing.

5) ICU and hospital length of stay (days).

6) ICU and hospital mortality.

7) 28‐day mortality.

### 2.5 Study Selection, Citation Management and Screening

All retrieved studies will be exported to Endnote 20 (Clarivate analytics). Citations will then be imported into Covidence systematic review platform (Veritas Health Innovation, Melbourne, Australia). Duplicates will be removed in Covidence. Titles and abstracts will be reviewed against our inclusion and exclusion criteria by two independent reviewers (PT and PS). Articles not meeting initial screening will be excluded, where there is disagreement between reviewers a third independent senior reviewer (B.J.) will make the final decision.

Articles included following title and abstract screening will then be reviewed in full against inclusion and exclusions criteria by two independent reviewers and discrepancies or conflicts resolved by a third senior reviewer. We will report the primary reason for article exclusion in Covidence and the process of study selection will be mapped by a PRISMA flowchart.

### 2.6 Data Extraction and Management

Data will be extracted in duplicate by two independent reviewers using a pre‐piloted standardized data extraction form. Data will be extracted in Covidence. Extracted data will include:

1) Study design, methodology, study type, study setting, study period, study authors, study location and study funding.

2) Characteristics of the included population, including, number of participants, age, sex, body mass index, intensive care unit admission diagnosis, co‐morbidities, APACHE II/disease severity scores.

3) Recruitment procedures.

4) Interventions including IMT and EMST methods, including measures of compliance with the intervention.

5) Primary outcome measures of included studies.

6) Secondary outcome measures of included studies.

7) Definitions of outcome measures of included studies.

8) Reported findings of included studies.

We will contact study authors for clarification of findings, methodology or missing data if required. We will discuss any missing data as a limitation of this review. Any inconsistencies in extracted data will be reviewed by a senior third reviewer and resolved by consensus.

### 2.7 Risk of Bias and Quality Assessment

Risk of bias will be assessed independently by two reviewers with any discrepancies to be decided by a third reviewer.

#### 2.7.1 Randomized Controlled Trials

To assess for bias in randomized studies, the Risk of Bias 2 tool (RoB 2) will be used, analyzing each study across its five domains. Studies will then be classified as: low risk of bias, some concerns or high risk of bias [31].

#### 2.7.2 Non‐Randomized Trials

For non‐randomized studies identified, we will use the Newcastle‐Ottowa scale (NOS). These studies will be analyzed across the tool's three domains of Selection, Comparability and Exposure (Case‐Control studies)/Outcome (Cohort and Cross‐sectional studies). We will then assign a “star” rating out of 8 or 9 depending on study type with “good” studies being rated 7 stars or higher.

We will use the Grading of Recommendations, Assessment, Development and Evaluation (GRADE) tool to assess the quality of evidence in each study. The certainty in evidence quality will be rated as follows: very low, low, moderate or high [32].

### 2.8 Statistical Analysis

If there is sufficient data, we will undertake meta‐analysis, and will report participant characteristics, setting, intervention, clinical outcomes, and methodological quality using evidence tables and will discuss our findings in the text. We will follow a sequential approach to data synthesis and will consider randomized controlled trials first, following by non‐randomized prospective and retrospective studies. We will report the methods used to evaluate swallowing and individual effectiveness of each method. We will record the number of patients experiencing the adverse outcomes (PED) and the number analyzed in each group. Where data permits, we will report odds ratios (OR) with 95% confidence intervals.

Secondary outcomes will be reported as a mix of dichotomous and continuous outcome data. We will present dichotomous data as number of participants experiencing the particular outcome and calculated OR with 95% confidence intervals. Continuous data will be presented as arithmetic means and standard deviation with 95% confidence intervals for each outcome. We will also extract and report medians and ranges where data permits.

We will assess data for meta‐analysis based upon the degree of clinical, statistical, and methodological heterogeneity between studies.

Where data from individual studies allows, we will pool reported results and present visually using Forest Plots. We will undertake a meta‐analysis using a random effects model to measure pooled estimates of effect. We will assess and report the degree of heterogeneity between studies using Cochran's *Q* test and the *I* ^2^ statistic. We will consider heterogeneity < 25% as low, 25%–75% as moderate and > 75% as high.

### 2.9 Subgroup Analysis

Heterogeneity between studies will be explored by subgroup analysis assessing factors such as study design, intervention, and participants. Proposed covariates will include: (1) severity of illness (graded by sequential organ failure assessment score or APACHEII score), (2) gender distribution (males vs. female), (3) type of training performed (EMST, EMST in combination with IMT, and EMST in combination with another training modality), (4) admission diagnosis (medical vs. surgical admission).

All data analysis will be undertaken using Cochrane Collaboration RevMan (Review Manager 2014) software.

## 3 Discussion

The etiology of PED is multifactorial, of which neuromuscular weakness is just one potential cause. Nevertheless, there is evidence from both healthy subjects and patients in non‐critically ill disease groups that EMST can induce both hypertrophy of the muscles of swallowing, and improvements in expiratory muscle size and strength, and a recent meta‐analysis suggests that such training can improve swallowing safety in non‐critically ill patients [29]. These changes could improve both swallowing and cough clearance and is a promising treatment for patients after liberation from mechanical ventilation.

This systematic review will aim to evaluate the current knowledge base and quality of evidence examining EMST in critically ill patients. We will identify and synthesize data from all available studies to assess whether there is a role for EMST across a range of primary and secondary outcomes in survivors of critical illness. We will also highlight areas in need of further research and help to guide current clinical practice in the application of expiratory muscle strength training to a critical care setting. Finally, the review will describe what methods of swallowing evaluation have been used in critical care research.

## Author Contributions

**Philip Skurok:** conceptualization, methodology, writing–original draft, writing–review and editing. **Brian W. Johnston:** conceptualization, methodology, writing–original draft, writing–review and editing, supervision. **Emma Brown:** methodology, resources. **Caroline Timothy:** methodology, resources. **Christopher Morse:** conceptualization, writing–review and editing, supervision. **Peter Turton:** conceptualization, methodology, writing–original draft, writing–review and editing, supervision.

All authors have read and approved the final version of the manuscript. B.J. had full access to all of the data in the study and takes complete responsibility for the integrity of the data and accuracy of the data analysis. B.J. registered the protocol.

## Ethics Statement

The authors have nothing to report.

## Consent

The authors have nothing to report.

## Conflicts of Interest

The authors declare no conflicts of interest.

## Transparency Statement

The lead author B. Johnston affirms that this manuscript is an honest, accurate, and transparent account of the study being reported; that no important aspects of the study have been omitted; and that any discrepancies from the study as planned (and, if relevant, registered) have been explained.

## Supporting information

Supporting information.

## Data Availability

The authors have nothing to report.
